# Editorial: Mining and utilization of favorable gene resources in rice

**DOI:** 10.3389/fpls.2023.1289069

**Published:** 2023-09-19

**Authors:** Ying Wang, Xueyong Li, Ryuji Ishikawa, Xiaojin Luo

**Affiliations:** ^1^ State Key Laboratory of Genetic Engineering and Engineering Research Center of Gene Technology (Ministry of Education), School of Life Sciences, Fudan University, Shanghai, China; ^2^ Ministry of Education Key Laboratory for Biodiversity Science and Ecological Engineering, Department of Ecology and Evolutionary Biology, School of Life Sciences, Fudan University, Shanghai, China; ^3^ National Key Facility for Crop Gene Resources and Genetic Improvement, Institute of Crop Sciences, Chinese Academy of Agricultural Sciences, Beijing, China; ^4^ Faculty of Agriculture and Life Science, Hirosaki University, Hirosaki, Aomori, Japan

**Keywords:** gene resources, germplasm utilization, yield component traits, mapping and cloning, genome-wide association study, molecular design breeding, rice

Agriculture has been evolving along with the development of human society. Agricultural germplasm resources play a crucial role in supporting the development of human civilization (Doebley et al., 2006; Tian et al., 2021). With the change of agricultural population structure, there are new requirements for germplasm resources (Yu and Li, 2021). Combined with global climate change, crops should also be able to cope with the challenges of environmental stresses and maintain a stable increase in production (Shahzad et al., 2021). Subsequently, rice has new breeding requirements in production, quality, and yield (Zeng et al., 2017). This Research Topic covers multiple approaches to exploit rice germplasm, excavate and utilize the key genes, and understand the molecular basis of yield component traits.

## Mapping and cloning of new genes or revealing new functions

The classical mapping and cloning technique still play an important role in the discovery of new genetic loci. Kim et al. cloned the *OsD2* gene by map-based cloning based on the previously reported quantitative trait locus (QTL) *qLTG1* responsible for low-temperature germinability in rice. Lan et al. identified the *hfr131* gene, which affects plant height and grain length in rice, through genetic analysis of introgression line. Yin et al. detected 16 QTL ranges for salt tolerance at bud stage in rice and a new candidate gene *LOC_Os12g25200*, using recombinant inbred line populations.

Interestingly, these works revealed new functions of known genes. *OsD2* is allelic to *DWARF2*, which encodes a cytochrome P450 involved in brassinosteroid (BR) biosynthesis. Although most alleles of the *dwarf2* mutant showed severe dwarfism with undesirable traits which reduced their breeding application value, the *OsD2* allele improved low-temperature germinability without any harmful phenotype. As a new allele of the BR receptor *OsBRI1*, *hfr131* affected rice plant height through the dn-type internode elongation pattern, which had not been reported previously in any BR biosynthesis- or *OsBRI1*-defective mutants. These findings suggest that the SNPs polymorphism may confer new functions to the known genes, and the discovery of new alleles is helpful to expand the diversity of gene function and mechanism.

## Genome-wide association study facilitates mining of loci associated with complex agronomic traits

Complex agronomic traits of crops involve complex genetic loci, and genome-wide association study (GWAS) can significantly improve the identification efficiency of potential QTLs. Li et al. conducted GWAS using 211 rice accessions to determine salt tolerance germinability indices. A total of 43 QTLs were identified, 18 of which were co-localized with previous studies. According to the RNA-seq and haplotype analysis, rice varieties with elite haplotypes in *LOC_Os03g13560*, *LOC_Os03g13840* and *LOC_Os03g14180* genes had high salt tolerance germinability. Dai et al. detected high-quality loci responsible for high seed vigor from 346 diverse accessions. By GWAS, 51 significant SNPs were identified, which were further validated using chromosome segment substitution lines. Integrating gene expression, gene annotation, and haplotype analysis, 21 strong candidate genes were identified. The functions of *LOC_Os01g11270* and *LOC_Os01g55240* were further verified by CRISPR/Cas9. Xu et al. combined GWAS and linkage mapping to analyze the candidate intervals for seedling salinity tolerance of 295 japonica rice varieties. After identifying the lead SNP (Chr12_20864157), the candidate gene *LOC_Os12g34450* was obtained by haplotype analysis, qRT-PCR, and sequence analysis.

Salt tolerant germinability, high seed vigor and seedling salinity tolerance correspond to the key problems affecting the yield of rice in agricultural production. By GWAS, these works verified the known genetic loci and found new functional genes, which provided a promising resource for solving the problems. In addition, the integration of multiple methods (chromosome segment substitution, linkage mapping, RNA-seq database, haplotype analysis, CRISPR/Cas9, etc.) can validate the results of GWAS and help identify candidate genes.

## Functional expansion of key gene resources via phylogenetic analysis and reverse genetics

Functional expansion of known key genes is another effective pathway for favorable gene resources. Papain-like cysteine proteases (PLCPs) play a crucial role in plant growth and development. Li et al. constructed CRISPR/Cas9 lines and showed that a PLCP, an oryzain alpha chain precursor (OCP), negatively regulated resistance to blast disease. OCP interacted with OsRACK1A or OsSNAP32 and influenced the expression of many resistance-related genes. Zheng et al.‘s work was based on the cell cycle-associated transcription factors E2F (E2 promoter binding factor), and their downstream target gene the mismatch repair-related gene *OsMSHs* (Mutated S homologue). They systematically categorized six rice E2Fs, and constructed four *msh* mutants using the CRISPR-Cas9 technique. This study elucidated the mechanism of *E2F* and *MSH* for enhancing cadmium stress tolerance in rice. Zeng et al. focused on the glutamate-like receptor (GLR) genes, which play a crucial role in signal transduction and communication. An integrated approach involving phylogenetic analysis, phenotypic and functional characterization and comprehensive population genetic analyses was employed to understand the functionalities of 26 rice *GLR* genes. The results suggested that natural variations at most rice *GLR* loci had potential value in improving the productivity and tolerance to abiotic stresses. These studies expand our understanding of these known key gene functions, and genes in related biological processes and molecular networks are also worthy for the further exploration of effective gene resources.

Finally, one review article from Zhong et al. presented a comprehensive review on the recent breakthroughs in rice yield traits and molecular design breeding in China. The authors believed that the further mining of genes and gene regulatory networks, development and utilization of molecular markers, establishment of the high-throughput and low-cost genotype detection system, and rational aggregation of high-quality genes (genes related to yield traits, stress resistance traits and quality traits) to breed new varieties with high yield and superior quality, will be an inevitable trend in future rice research.

## Perspectives

In summary, this Research Topic brought recent advances in the mining and utilization of rice germplasm resource. These studies indicated the new situations in the exploration of favorable gene resources. For example, crop yield breeding needs to balance the influence of environmental stress, and molecular design breeding needs to consider the pleiotropy between genes and complex regulatory networks among genes. Overcoming these problems will open the way to achieve a further breakthrough in the current yield level.

## Author contributions

YW: Writing – original draft. XLi: Writing – review & editing. RI: Writing – review & editing. XLu: Writing – review & editing.
